# Dysregulation of piRNA biogenesis-associated proteins in cancer: mechanisms, evidence, and translational constraints

**DOI:** 10.3389/fmolb.2026.1906635

**Published:** 2026-07-29

**Authors:** Siyun Peng, Bo Wei, Xing Li, Ermao Li

**Affiliations:** 1 Hengyang Medical College, Institute of Translational Medicine, University of South China, Hengyang, Hunan, China; 2 Gansu Provincial Hospital of Traditional Chinese Medicine, Lanzhou, Gansu, China

**Keywords:** piRNA, PIWI proteins, piRNA biogenesis, RNA network, non-coding RNA, cancer, DDX4, PLD6

## Abstract

PIWI-interacting RNAs (piRNAs), PIWI proteins, and piRNA biogenesis-associated proteins form a conserved RNA network that protects germline genome integrity and is increasingly linked to cancer biology. Most cancer reviews have emphasized altered piRNA abundance or PIWI expression, whereas the upstream machinery that generates, loads, trims, and stabilizes piRNAs has received less critical attention. For the RNA Networks and Biology section, this Review re-evaluates piRNA biogenesis proteins as RNA-network regulators rather than as isolated tumor biomarkers. We summarize the biochemical logic of precursor selection, mitochondrial processing, PIWI loading, 3-end maturation, and effector complex assembly, focusing on PIWIL1-4, PLD6/MitoPLD, MOV10L1, DDX4/VASA, GASZ, TDRKH, PNLDC1, HENMT1, Tudor-domain proteins, HSP90, and MAEL. We then assess cancer evidence by distinguishing direct perturbation studies from expression associations, animal-germline mechanisms, and computational inference. Current evidence supports tumor-context-specific roles for PIWIL1, PIWIL2, DDX4, TDRD1, MAEL, and emerging PLD6 biology in selected tumors, whereas several canonical maturation or scaffold proteins remain underexplored in human cancer. We propose that dysregulated piRNA biogenesis proteins may influence cancer through piRNA-dependent small-RNA imbalance and piRNA-independent reactivation of germline RNA-processing, epigenetic, metabolic, and immune programs. Finally, we discuss biomarker and therapeutic opportunities while emphasizing unresolved controversies, technical limitations in piRNA detection, reproductive toxicity, and the need for causal validation in human tumor models.

## 1 Introduction

piRNAs are PIWI-bound small non-coding RNAs that were originally defined by their ability to repress transposable elements in animal germ cells (Aravin et al., 2007; Siomi et al., 2011; Czech and Hannon, 2016; Wang et al., 2023). This canonical function remains central to the field: disruption of piRNA pathway proteins frequently causes meiotic arrest, germ-cell loss, transposon derepression, and infertility in model organisms (Watanabe et al., 2011; Fu et al., 2016; Ding et al., 2017; Nishimura et al., 2018). The pathway is therefore best understood as an RNA network that couples precursor transcription, mitochondrial processing, PIWI loading, small-RNA maturation, and effector-complex assembly.

Cancer studies have broadened the scope of this pathway by reporting abnormal piRNA expression and ectopic PIWI-family protein expression in multiple tumor types (Cai et al., 2022; Chattopadhyay et al., 2022; Limanówka et al., 2024). These observations are plausible because tumor cells often reactivate developmental and germline programs, remodel chromatin, alter RNA processing, and acquire stem-like states. However, the field still faces two conceptual problems. First, changes in piRNA abundance are often interpreted without testing whether the upstream biogenesis machinery is altered. Second, expression associations are sometimes written as causal cancer mechanisms even when the supporting evidence comes from bulk transcriptomic data, small cohorts, or germline animal models.

This Review addresses these issues from the perspective of RNA Networks and Biology. We ask which protein modules define the piRNA biogenesis network, how they are repurposed or dysregulated in cancer, and which translational claims are currently supported. We use piRNA biogenesis-associated proteins broadly to include canonical pathway components and auxiliary factors that regulate precursor processing, PIWI loading, piRNA maturation, or PIWI-associated effector functions. We deliberately separate direct human cancer perturbation evidence from patient-cohort association, computational inference, and animal-germline mechanism. This evidence hierarchy is essential for a field in which conserved molecular mechanisms are informative but not automatically transferable across species, cell type, or disease state.

## 2 Biochemical framework of the piRNA biogenesis network

### 2.1 Precursor processing and mitochondrial organization

**FIGURE 1:**
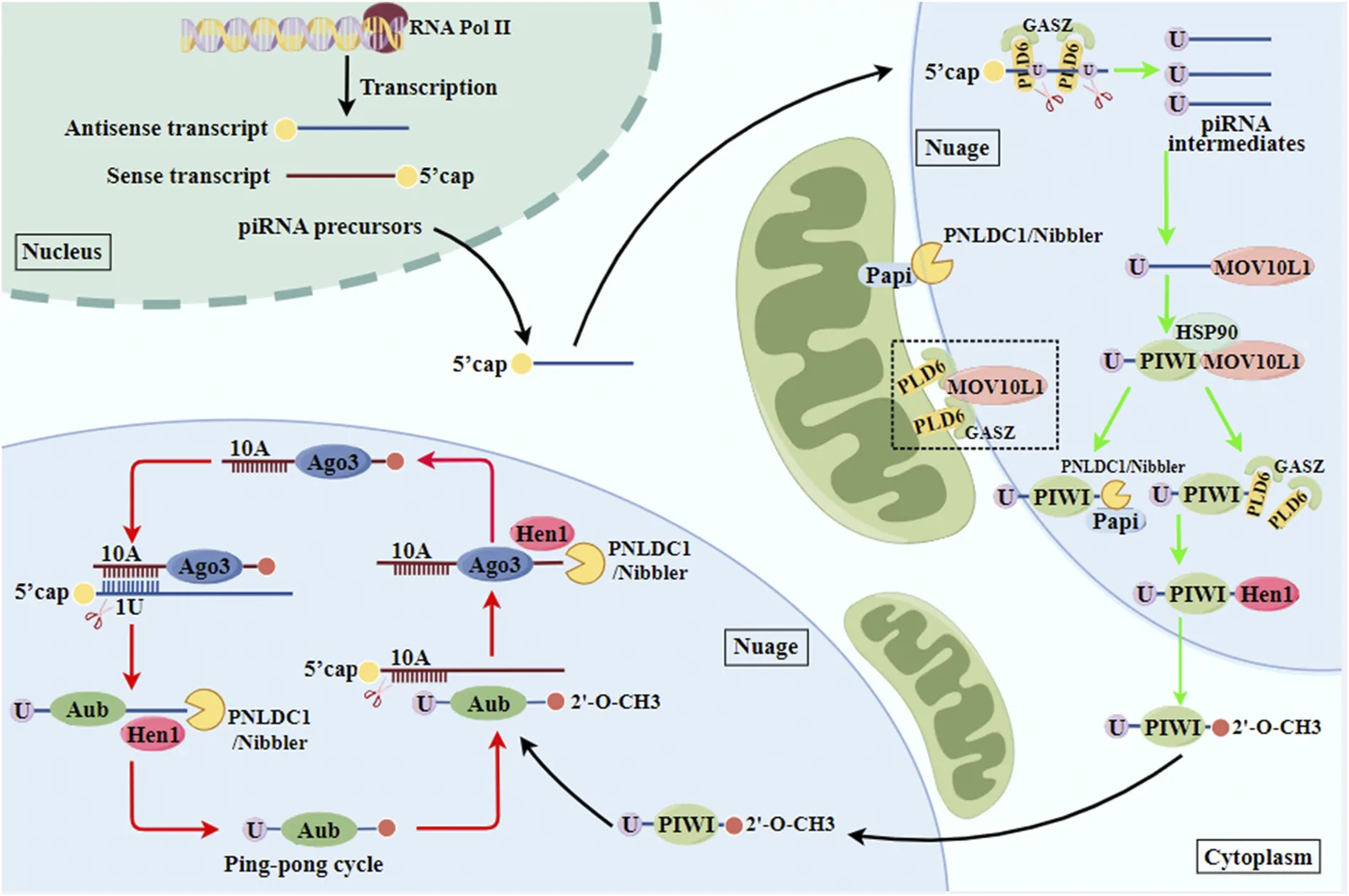
Core piRNA biogenesis modules and species boundaries relevant to cancer interpretation. Long piRNA precursors are transcribed from piRNA clusters and transported to cytoplasmic or perinuclear processing compartments. PLD6\MitoPLD, MOV10L1, and GASZ contribute to precursor engagement and 5-end formation at mitochondria-associated sites. PIWI proteins load piRNA intermediates through conserved PAZ and PIWI domains, whereas TDRKH\PNLDC1 and HENMT1 regulate 3-end trimming and methylation. DDX4\VASA, Tudor-domain factors, and HSP90-related chaperones support granule organization, ping-pong amplification, and complex stability. The figure includes canonical model-organism components to illustrate the pathway logic; direct transfer to human cancer requires confirmation of the expressed PIWI paralogue, protein localization, small-RNA partner, and tumor-cell dependency. By Figdraw. (Figdraw licence https://www.figdraw.com/).

Canonical piRNA biogenesis is spatially organized rather than a simple linear enzyme cascade (Figure 1). In animals, long piRNA precursors arise from genomic piRNA clusters and are transported to cytoplasmic or perinuclear granules, where they encounter mitochondrial processing factors and PIWI proteins (Iwasaki et al., 2015; Czech and Hannon, 2016). PLD6, also known as MitoPLD in mice and related to Zucchini in *Drosophila*, contributes to precursor cleavage and 5-end formation (Watanabe et al., 2011; Mohn et al., 2015). MOV10L1 binds precursor RNAs and resolves secondary structures, including G-quadruplex-containing regions, thereby supporting precursor entry into the processing pathway (Vourekas et al., 2015; Zhang et al., 2019). GASZ acts as a mitochondrial scaffold that helps recruit processing factors to outer mitochondrial membrane-associated sites (Munafò et al., 2019; Zhang et al., 2016).

### 2.2 PIWI loading, 3-end maturation, and amplification

PIWI proteins bind piRNA intermediates through conserved Argonaute-family domains. The PAZ domain anchors the piRNA 3 end, whereas the PIWI domain adopts an RNase H-like fold and mediates Slicer activity in catalytically competent PIWI proteins (Juliano et al., 2011; Gainetdinov et al., 2023). Mature length arises through 3-to-5 trimming, involving PNLDC1/Nibbler and the adaptor protein TDRKH/Papi in several systems (Saxe et al., 2013; Hayashi et al., 2016; Ding et al., 2017). HENMT1/HEN1 then catalyzes 2-O-methylation at the 3 end, protecting mature piRNAs from degradation (Hempfling et al., 2017; Kaldis and Zhao, 2024). In ping-pong amplification, one PIWI-piRNA complex cleaves a complementary transcript and creates the 5 end of a secondary piRNA. Reciprocal cleavage generates the characteristic 10-nt 5-overlap and nucleotide-bias signatures used to infer amplification (Aravin et al., 2007; Czech and Hannon, 2016). DDX4/VASA, Tudor-domain proteins, and HSP90-related chaperone systems help maintain granule organization and complex stability (Karam et al., 2017; Ichiyanagi et al., 2014; Adashev et al., 2024). In *Drosophila*, Aubergine and Ago3 execute the cytoplasmic ping-pong loop, whereas mammalian spermatogenesis uses stage- and compartment-specific PIWI paralogues. Human PIWIL1-4 should therefore not be treated as direct one-to-one substitutes for animal PIWI proteins in tumors.

### 2.3 Species boundaries, PIWI paralogues, and cancer interpretation

The conservation of PIWI, PLD6, MOV10L1, TDRKH, and related proteins supports the use of model organisms to define biochemical principles (Konstantinidou et al., 2024; Lv et al., 2023). This conservation is modular rather than identical. *Drosophila* Piwi, Aubergine, and Ago3 partition nuclear silencing and cytoplasmic ping-pong functions. Mouse PIWI paralogues act at different stages of spermatogenesis and differ in localization, timing, and small-RNA partners. Humans encode PIWIL1, PIWIL2, PIWIL3, and PIWIL4, but their expression patterns and pathway competence in somatic tumors are less completely resolved. These species and stage boundaries are central to cancer interpretation. Conservation does not prove that the same protein executes the same pathway in a cancer cell. A protein required for spermatogenesis may be ectopically expressed in tumors, but the tumor context may repurpose only part of its germline function. This is particularly relevant for PIWIL1, PIWIL2, DDX4, TDRD1, and MAEL, for which cancer studies often report effects on chromatin, signaling, alternative splicing, metabolism, or stemness without complete demonstration of piRNA dependence (Huang et al., 2021; Wang et al., 2021; Kim et al., 2023; Olotu et al., 2024) (Figure 1).

## 3 Physiological baseline and cancer-relevant boundaries

Most piRNA biogenesis-associated proteins are enriched in germ cells, where they protect genome integrity during gametogenesis. This restricted physiological context is central to cancer interpretation. Ectopic expression in tumors may create a cancer-testis vulnerability, but systemic inhibition may affect fertility or stem-cell compartments. PIWIL1, PIWIL2, and PIWIL4 contribute to germline piRNA function, transposon repression, and developmental regulation in a species- and stage-dependent manner (Juliano et al., 2011; Zhang et al., 2021; Wang et al., 2022; Lv et al., 2023). MOV10L1 is a germ-cell-enriched helicase required for piRNA precursor processing and transposon silencing (Vourekas et al., 2015; Fu et al., 2016; Zhang et al., 2019). PLD6 and GASZ are linked to mitochondrial processing platforms, whereas TDRKH, PNLDC1, and HENMT1 define the 3-end maturation layer (Saxe et al., 2013; Watanabe et al., 2011; Ding et al., 2017; Nishimura et al., 2018; Kaldis and Zhao, 2024).

The biological basis of germline-tumor expression is unlikely to be uniform. Some tumors may show passive epigenetic leakage, in which global chromatin disruption, DNA hypomethylation, or lineage infidelity permits germline genes to become detectable. Other tumors may actively select germline-program components when they improve stem-like growth, genome plasticity, RNA granule organization, immune escape, or stress tolerance. These alternatives have different evidentiary requirements. Passive leakage can be supported by broad co-expression of cancer-testis genes. Active selection requires tumor-cell perturbation, rescue experiments, localization data, and matched small-RNA or RNA-processing readouts. The review therefore treats ectopic expression as a starting observation rather than proof of pathway activity.

Several factors commonly discussed with the piRNA pathway have broader cellular functions. HSP90 is a general cancer chaperone with many clients, so effects of HSP90 inhibitors cannot be attributed to piRNA biogenesis without pathway-specific assays (Birbo et al., 2021; Li and Luo, 2023). TDRD1 is a Tudor-domain cancer-testis antigen that can regulate prostate cancer proliferation through snRNP biogenesis rather than canonical piRNA processing (Kim et al., 2023). MAEL is a germline granule-associated protein linked to cancer cell survival, drug resistance, and metabolic remodeling (Zhang et al., 2017; Shi et al., 2022; Zhou et al., 2023). These examples explain why germline pathway maps should guide cancer hypotheses but should not replace direct tumor validation. They also motivate the separation of piRNA-dependent functions from piRNA-independent or pleiotropic activities in the evidence framework below (Table 1).

**TABLE 1:** Canonical functions and cancer-relevant evidence for piRNA biogenesis-associated proteins.

Protein/module	Canonical RNA-network role	Cancer-relevant evidence	Main limitations	References
PIWIL1-4	PIWI-piRNA effector function, transposon control, and developmental regulation	Strongest for selected PIWIL1/2 contexts; PIWIL4 less resolved	Tumor expression does not prove active piRNA biogenesis	Juliano et al. (2011), Zhang et al. (2021), Wang et al. (2022), Lv et al. (2023), Huang et al. (2021), Wang et al. (2021)
MOV10L1	Precursor binding and RNA structure resolution	Mainly expression signatures or computational associations in cancer	Germline mechanism may be overextended	Vourekas et al. (2015), Fu et al. (2016), Zhang et al. (2019), Wu et al. (2020)
DDX4/VASA	RNA helicase and germ-granule organization	Emerging direct cancer evidence for ribonucleoprotein granules and xenograft growth	Mechanism may be RNA-granule biology rather than piRNA dependence	Adashev et al. (2024), Hashimoto et al. (2008), Chen et al. (2018), Olotu et al. (2024)
PLD6/GASZ	Mitochondrial processing and 5-end formation	Emerging PLD6 colorectal cancer evidence; GASZ cancer evidence limited	Metabolic and piRNA functions must be separated	Watanabe et al. (2011), Zhang et al. (2016), Miao et al. (2024), Lee et al. (2025)
TDRKH/PNLDC1/HENMT1	3-end trimming, methylation, and piRNA stability	HENMT1 associations in selected cancer datasets	Direct piRNA methylation dependency remains untested	Saxe et al. (2013), Ding et al. (2017), Nishimura et al. (2018), Kaldis and Zhao (2024), Reyimu et al. (2023)
TDRD1/HSP90/MAEL	Complex assembly, chaperoning, or germline-program regulation	TDRD1 and MAEL have functional cancer evidence	Broad functions may be mislabeled as piRNA biogenesis	Ichiyanagi et al. (2014), Karam et al. (2017), Kim et al. (2023), Zhang et al. (2017), Shi et al. (2022), Zhou et al. (2023)

## 4 Cancer evidence by RNA-network module

### 4.1 PIWI proteins

PIWI proteins are the most frequently investigated cancer-associated members of the piRNA pathway, but literature frequency should not be equated with pathway-level functional centrality. PIWIL1 has been linked to aggressive features in gastric cancer, glioblastoma, and hepatocellular carcinoma, with proposed connections to PI3K/AKT/mTOR signaling, glioma stem-cell maintenance, lipid metabolism, and immunosuppressive microenvironmental crosstalk (Shi et al., 2020; Huang et al., 2021; Wang et al., 2021). These data support PIWIL1 as a tumor-context-specific cancer regulator, but they do not establish that all PIWIL1-driven phenotypes are canonical piRNA-biogenesis events.

PIWIL2 has been reported in cervical, colorectal, and breast cancer contexts and may influence invasion, p53-p21 signaling, STAT3/SRC signaling, and methylation-linked repression of tumor suppressor genes (Ponnusamy et al., 2017; Cai et al., 2022). However, PIWIL2 biology is strongly context- and isoform-dependent. A conservative framing is that PIWIL2 represents a cancer-testis RNA-network factor with tumor-type-specific functions, not a uniform oncogenic biomarker.

### 4.2 RNA helicases and germ-granule factors

MOV10L1 has a well-supported role in germline precursor processing, but its cancer evidence remains comparatively weak. Transcriptomic studies have identified MOV10L1 in RNA-binding-protein-related prognostic signatures, including clear-cell renal cell carcinoma (Wu et al., 2020). Such findings are useful for hypothesis generation but should not be presented as mechanistic proof without tumor-cell perturbation, rescue experiments, and matched piRNA profiling.

DDX4/VASA has more substantial emerging evidence. Earlier ovarian cancer studies reported DDX4 expression and potential links to checkpoint or stemness-associated features (Hashimoto et al., 2008; Chen et al., 2018). More recently, DDX4 was shown to form cytoplasmic germline-like ribonucleoprotein granules in cancer cells, and DDX4 deletion altered transcriptomes, affected splicing, and impaired xenograft tumor growth (Olotu et al., 2024). Together, these data place DDX4 among cancer-germline RNA-network factors. However, current data point more directly to granule and RNA-processing biology than to a fully demonstrated piRNA-dependent mechanism.

### 4.3 Mitochondrial processing proteins

PLD6/MitoPLD links mitochondrial biology to piRNA precursor processing. In germ cells, PLD6 disruption impairs piRNA production and transposon control (Watanabe et al., 2011). Recent colorectal cancer evidence reported that PLD6 promotes Wnt/beta-catenin-associated tumorigenic phenotypes through mitochondrial metabolic reprogramming (Lee et al., 2025). These findings sharpen the cancer rationale for PLD6, but the piRNA-dependent fraction of this phenotype remains to be clarified. GASZ is a mechanistically important scaffold for mitochondrial piRNA processing and germ-cell development, but its direct cancer evidence remains limited to antigenicity or expression-level observations (Zheng et al., 2011; Miao et al., 2024).

### 4.4 Maturation enzymes and auxiliary factors

**FIGURE 2:**
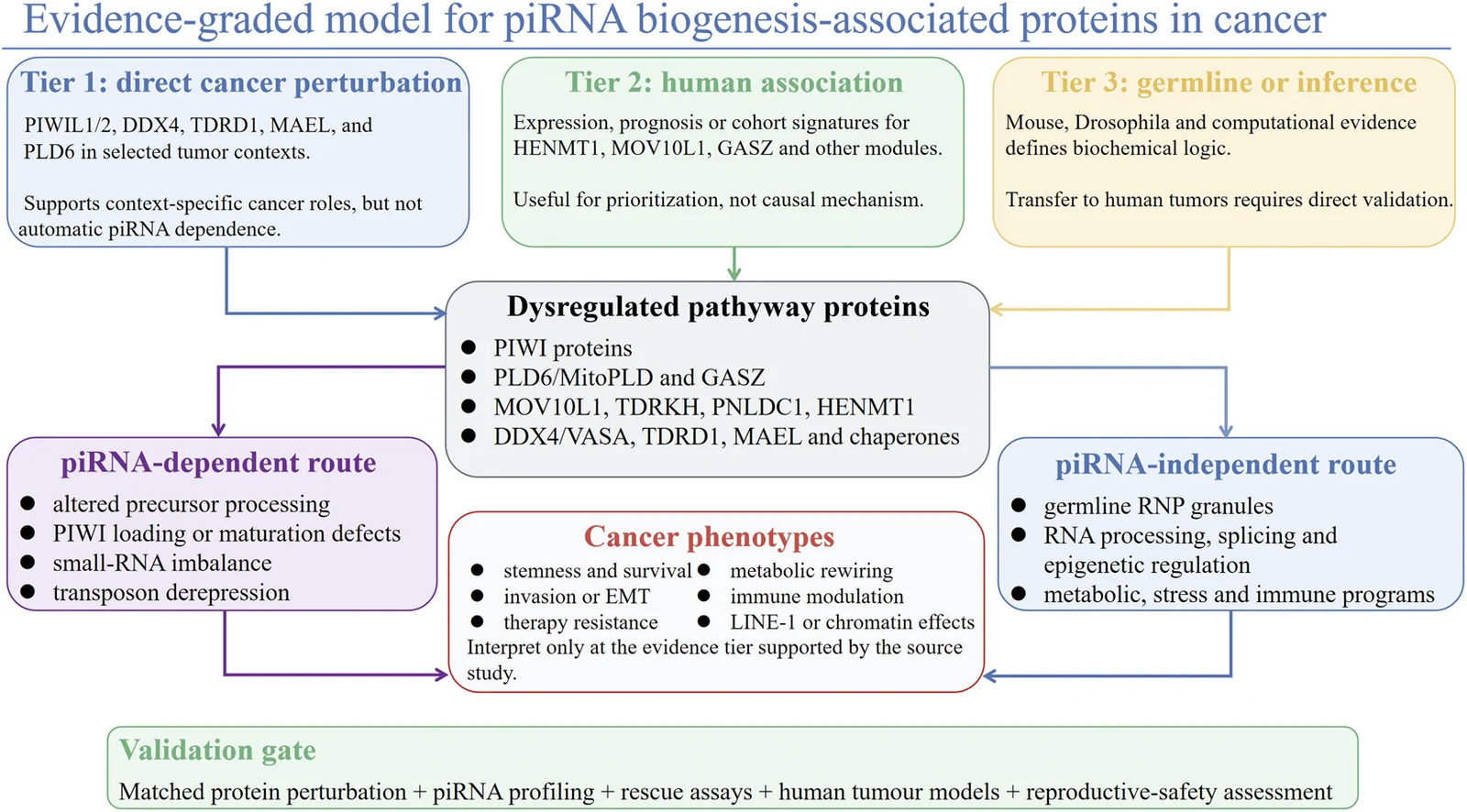
Evidence-graded model linking dysregulated piRNA biogenesis-associated proteins to cancer phenotypes. Tier 1 evidence includes direct tumor-cell perturbation or functional cancer-model data; Tier 2 evidence includes human expression, prognosis, or cohort associations; Tier 3 evidence includes germline model biology or computational inference. Dysregulated proteins may affect cancer through piRNA-dependent routes, including altered processing, PIWI loading, maturation, small-RNA imbalance, and transposon control, or through piRNA-independent routes, including germline RNP granules, RNA processing, splicing, epigenetic regulation, metabolic stress programs, and immune modulation. Each cancer phenotype should be interpreted only at the evidence tier supported by the source study.

TDRKH, PNLDC1, and HENMT1 define the 3-end maturation layer of canonical piRNA biology. Model systems show that their disruption can reduce mature piRNA abundance, alter piRNA length or methylation, and derepress transposable elements (Saxe et al., 2013; Ding et al., 2017; Nishimura et al., 2018; Kaldis and Zhao, 2024). In cancer, HENMT1 has been associated with esophageal cancer expression signatures and prognosis-related features (Reyimu et al., 2023). These observations warrant further study but should remain association-level claims until direct piRNA methylation and functional dependency assays are performed. MAEL has broader cancer relevance as a germline-program factor. It has been linked to gastric cancer progression, hepatocellular carcinoma stemness and sorafenib resistance, breast cancer metabolic reprogramming, and bladder cancer invasion through mechanisms involving ILKAP degradation, PTGS2/AKT/STAT3 signaling, chaperone-mediated autophagy, and DNMT3B-dependent epigenetic regulation (Zhang et al., 2017; Shi et al., 2022; Zhou et al., 2023; Li et al., 2016). MAEL should therefore be interpreted as a broader germline-program factor rather than as a dedicated piRNA biogenesis enzyme (Figure 2; Table 2).

**TABLE 2:** Cancer-associated evidence, functional interpretation, and evidence tier for piRNA biogenesis-associated proteins.

Protein	Cancer context	Current support	Current functional interpretation	Evidence tier	References
PIWIL1	Gastric cancer, glioblastoma, hepatocellular carcinoma	Functional and association evidence in selected models	Candidate context-specific regulator	Tier 1, functional plus association	Shi et al. (2020), Huang et al. (2021), Wang et al. (2021)
PIWIL2	Cervical cancer, colorectal cancer, breast cancer	Mixed functional and isoform-dependent evidence	Cancer-testis RNA-network factor	Tier 1-2, mixed functional and isoform-dependent	Ponnusamy et al. (2017), Cai et al. (2022), Lu et al. (2022)
MOV10L1	Renal cancer datasets	Computational or expression association	Hypothesis-generating only	Tier 2-3, association with germline support	Wu et al. (2020), Vourekas et al. (2015), Fu et al. (2016), Zhang et al. (2019)
DDX4/VASA	Ovarian cancer	Emerging direct cancer-granule evidence	RNA-processing/granule factor; piRNA dependence unresolved	Tier 1, emerging direct cancer-granule evidence	Hashimoto et al. (2008), Chen et al. (2018), Olotu et al. (2024)
PLD6	Colorectal cancer; ovarian cancer	Emerging mechanistic evidence	Promising but piRNA linkage requires testing	Tier 1-2, emerging metabolic evidence	Watanabe et al. (2011), Lee et al. (2025)
HENMT1	Esophageal and ovarian cancer	Expression/prognosis associations	Maturation enzyme with unproven cancer dependency	Tier 2, expression and prognosis association	Hempfling et al. (2017), Reyimu et al. (2023), Kaldis and Zhao (2024)
TDRD1	Prostate cancer	Functional evidence through small nuclear ribonucleoprotein biogenesis	Non-canonical RNA-processing mechanism	Tier 1, functional non-canonical RNA-processing evidence	Kim et al. (2023)
MAEL	Gastric cancer, breast cancer, bladder cancer, hepatocellular carcinoma	Functional evidence across several models	Germline-program factor, not only piRNA biogenesis	Tier 1, functional evidence in several models	Li et al. (2016), Zhang et al. (2017), Shi et al. (2022), Zhou et al. (2023)

Evidence tier definitions: Tier1, direct functional evidence from perturbation studies in cancer models; Tier 2, evidence from human association or expression studies; Tier3, indirect evidence inferred from germline studies or computational analyses.

## 5 Translational opportunities and constraints

The piRNA biogenesis network is attractive for translation because several components are normally enriched in germ cells and become ectopically expressed in tumors. This creates a plausible cancer-testis therapeutic window. At present, however, the field is closer to target discovery than to clinical implementation. Experimentally supported candidates include anti-PL2L60 antibodies targeting a PIWIL2-related splice variant, MAEL-axis inhibition in sorafenib-resistant hepatocellular carcinoma models, DDX4 perturbation in cancer-germline granule models, and PLD6 biology in colorectal cancer (Lu et al., 2022; Shi et al., 2022; Olotu et al., 2024; Lee et al., 2025).

The main constraints are mechanistic specificity, reproductive toxicity, tumor heterogeneity, and detection technology. PIWI proteins share structural logic with Argonaute-family proteins, raising selectivity concerns for small molecules (Ponnusamy et al., 2017; Cai et al., 2022). Many pathway proteins are essential for spermatogenesis, so systemic inhibition may affect fertility. Tumor expression may also be confined to stem-like subpopulations or vary under therapy pressure. Finally, piRNA detection remains technically challenging because of short length, 3-end modifications, repetitive genomic origin, and imperfect annotation. Newer single-cell, multi-omics, and direct RNA sequencing tools may improve resolution, but they should be used to test specific hypotheses rather than to overstate clinical readiness (Song et al., 2025; Zhou et al., 2025; Cao and Gao, 2022). For technical considerations, see Box 1

Box 1 **Technical considerations for interpreting tumor piRNA studies.**

Tumor piRNA profiling requires method-specific caution. Standard small-RNA sequencing can under-represent 3-end-modified piRNAs because adapter ligation and reverse transcription are biased by RNA end chemistry. Periodate oxidation or beta-elimination strategies can enrich 2-O-methylated small RNAs, but they do not by themselves prove PIWI loading or pathway activity. Size selection should be combined with stringent removal of miRNA, tRNA, rRNA, snoRNA, and degradation-fragment contamination. Reads from repetitive loci should be mapped with transparent multi-mapping rules. Stronger cancer claims require matched protein expression, PIWI immunoprecipitation or localization, piRNA length and sequence signatures, and perturbation-rescue experiments.

(Table 3).

**TABLE 3:** Therapeutic strategies targeting piRNA biogenesis-associated proteins: current status and translational roadmaps.

Strategy	Target/module	Current status	Key supporting evidence	Required validation	Primary translational barrier	References
Anti-PL2L60 antibody	PIWIL2-related splice variant	Preclinical	Anti-PL2L60 mAb (KAO3) exerts broad-spectrum anti-tumor activity, effectively inhibiting proliferation and invasion across multiple cancer models	Antigen specificity, normal-tissue expression, toxicity	Germline tissue on-target toxicity risk, absence of immunocompetent safety data and predictive biomarkers	Lu et al. (2022)
MAEL-axis targeting	MAEL/PTGS2/ AKT/STAT3 or metabolic pathways	Preclinical target biology	MAEL drives HCC stemness and sorafenib resistance via PTGS2 axis, MAEL inhibition combined with sorafenib exhibits synergistic therapeutic potential	Drug modality and patient-selection biomarkers	Absence of clinically actionable MAEL inhibitors, PTGS2 pathway pleiotropy limits tumor selectivity, lack of validated predictive biomarkers for patient stratification	Shi et al. (2022)
DDX4 disruption	Cancer-germline RNP granules	Emerging target biology	DDX4 forms oncogenic cytoplasmic granules; helicase activity drives tumor growth as a druggable candidate	Tumor selectivity and druggability	Germline on-target toxicity; poor helicase druggability; no validated patient stratification biomarkers	Olotu et al. (2024)
PLD6 pathway targeting	Mitochondrial metabolism and Wnt/beta-catenin	Early preclinical	PLD6 drives colorectal cancer tumorigenesis via mitochondrial metabolism and Wnt/beta-catenin activation	piRNA-dependent vs metabolic mechanism	Phospholipase family selectivity risk; mechanistic pleiotropy; no validated predictive biomarkers	Lee et al. (2025)
PIWI-domain inhibitors	PAZ/PIWI interface	Concept stage	In silico-designed inhibitors target PAZ/PIWI interface, preliminary *in vitro* binding and activity validation	Selectivity over AGO proteins and fertility safety	Argonaute family selectivity challenge; reproductive safety risk; limited *in vivo* pharmacology data	Lv et al. (2023)
HENMT1 blockade	3-end piRNA methylation	Concept stage	HENMT1 maintains piRNA stability via 3′-end methylation, genetic knockdown suppresses tumor malignancy *in vitro*	Direct cancer dependency and small-RNA toxicity	Broad small-RNA substrate off-target risk; reproductive safety concern; no targeted agents available	Nishimura et al. (2018), Reyimu et al. (2023)

Abbreviations: PL2L60, PIWIL2-like (PL2L) protein 60; mAb, monoclonal antibody; HCC, hepatocellular carcinoma; RNP, ribonucleoprotein.

## 6 Discussion

The strongest version of this manuscript is a balanced Review rather than a therapeutic-positioning article. The central contribution is not that piRNA biogenesis proteins are ready-made cancer drug targets, but that they form an RNA-network framework through which cancer cells may co-opt germline small-RNA, RNA-processing, and granule programs. This framing directly addresses current controversies: whether tumor-associated PIWI proteins act through piRNAs or through piRNA-independent functions; whether germline model mechanisms can be transferred to human cancer; and whether expression associations are sufficient for biomarker claims. The revised model in Figure 2 makes this logic explicit by linking each proposed phenotype to the level of evidence that supports it.

Future work should move beyond expression catalogs and address several testable questions. First, do oncogenic signaling pathways directly regulate PLD6, GASZ, PIWI proteins, or Tudor-domain factors through phosphorylation, altered localization, or complex assembly in tumor cells? Second, do cancer stress granules interact with piRNA-processing or germline RNP complexes, and can this interaction explain therapy resistance or stem-like states? Third, which tumor phenotypes persist after rescue with PIWI mutants that separate Slicer activity, piRNA binding, and non-canonical protein interactions? Fourth, does ectopic pathway expression reflect passive epigenetic leakage, active selection, or both in defined tumor states? Priority experiments include paired protein perturbation and piRNA profiling, rescue studies separating piRNA-dependent from piRNA-independent functions, human tumor organoid and patient-derived xenograft validation, antibody and localization controls for biomarker studies, and fertility or stem-cell toxicity assessment for therapeutic development. Until these gaps are addressed, PIWIL1, PIWIL2, DDX4, TDRD1, MAEL, and PLD6 are best described as promising but context-dependent candidates, whereas MOV10L1, GASZ, TDRKH, PNLDC1, and HENMT1 remain mechanistically important but insufficiently validated in human tumors.
